# Simulation data for the estimation of numerical constants for approximating pairwise evolutionary distances between amino acid sequences

**DOI:** 10.1016/j.dib.2019.104212

**Published:** 2019-07-08

**Authors:** Thomas Bigot, Julien Guglielmini, Alexis Criscuolo

**Affiliations:** Hub de Bioinformatique et Biostatistique ‒ Département Biologie Computationnelle, Institut Pasteur, USR 3756 CNRS, Paris, France

**Keywords:** Amino acid, Evolutionary model, Corrected distance, Uncorrected distance, Computer simulation, Nonlinear regression

## Abstract

Estimating the number of substitution events per site that have occurred during the evolution of a pair of amino acid sequences is a common task in phylogenetics and comparative genomics that often requires quite slow maximum-likelihood procedures when taking into account explicit evolutionary models. Data presented in this article are large sets of numbers of substitution events and associated numbers of observed differences between pairs of aligned amino acid sequences that have been generated through a simulation procedure of sequence evolution under a broad range of evolutionary models. These data are available at https://zenodo.org/record/2653704 (doi:10.5281/zenodo.2653704). They are accompanied in this paper by figures showing the strong relationship between the corresponding evolutionary and uncorrected distances, as well as estimated numerical constants that determine non-linear functions that fit the simulated data. These numerical constants can be useful to quickly estimate pairwise evolutionary distances directly from uncorrected distances between aligned amino acid sequences.

**Table:** Specifications table

Subject area	Computational biology, Bioinformatics
More specific subject area	Phylogenetics
Type of data	Text files, Images, Tables
How data was acquired	Computer simulation, nonlinear regression
Data format	Simulated, Analyzed
Experimental factors	Simulated data from publicly available phylogenetic trees
Experimental features	Amino acid sequence evolution simulation, evolutionary and uncorrected distance estimations, and nonlinear regression
Data source location	Institut Pasteur, Paris, France
Data accessibility	Simulation data and scatter plot figures are available at https://zenodo.org/record/2653704 (doi:10.5281/zenodo.2653704), numerical data and graphical representations of the nonlinear fitting are with this article

**Table:** 

**Value of the data** • The data proposed here should aim at enhancing the estimation of pairwise evolutionary distances between any pairs of amino acid sequences from a methodological, practical or educational point of view. • Available simulated data can be used to develop new methods and algorithms for more accurate or faster estimates of pairwise evolutionary distances. • Numerical data can be used to perform faster evolutionary distance estimates directly from the proportion of observed differences. • Associated figures can be used for educational purposes to illustrate the strong relationship between evolutionary and uncorrected distances.

## 1 Data

**Fig. 1:**
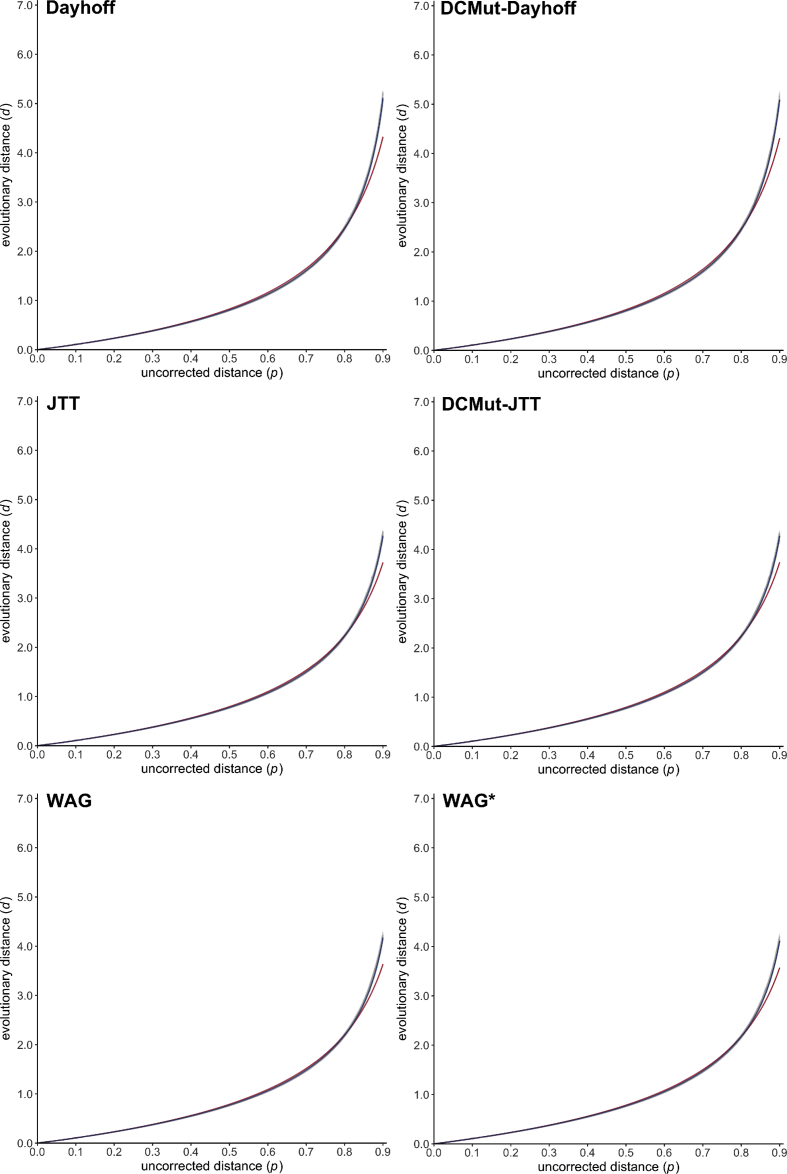
Scatter plots (gray dots) representing the relationship between the uncorrected distance *p* (x-axis) and the evolutionary distance *d* (y-axis) for the three general amino acid substitution models Dayhoff [1], JTT [18] and WAG [23] (left), and for their variants DCMut-Dayhoff, DCMut-JTT [26] and WAG* [23] (right). Estimated Poisson correction (PC) and equal-input (EI) gamma distance functions are drawn in red and blue, respectively.

**Fig. 2:**
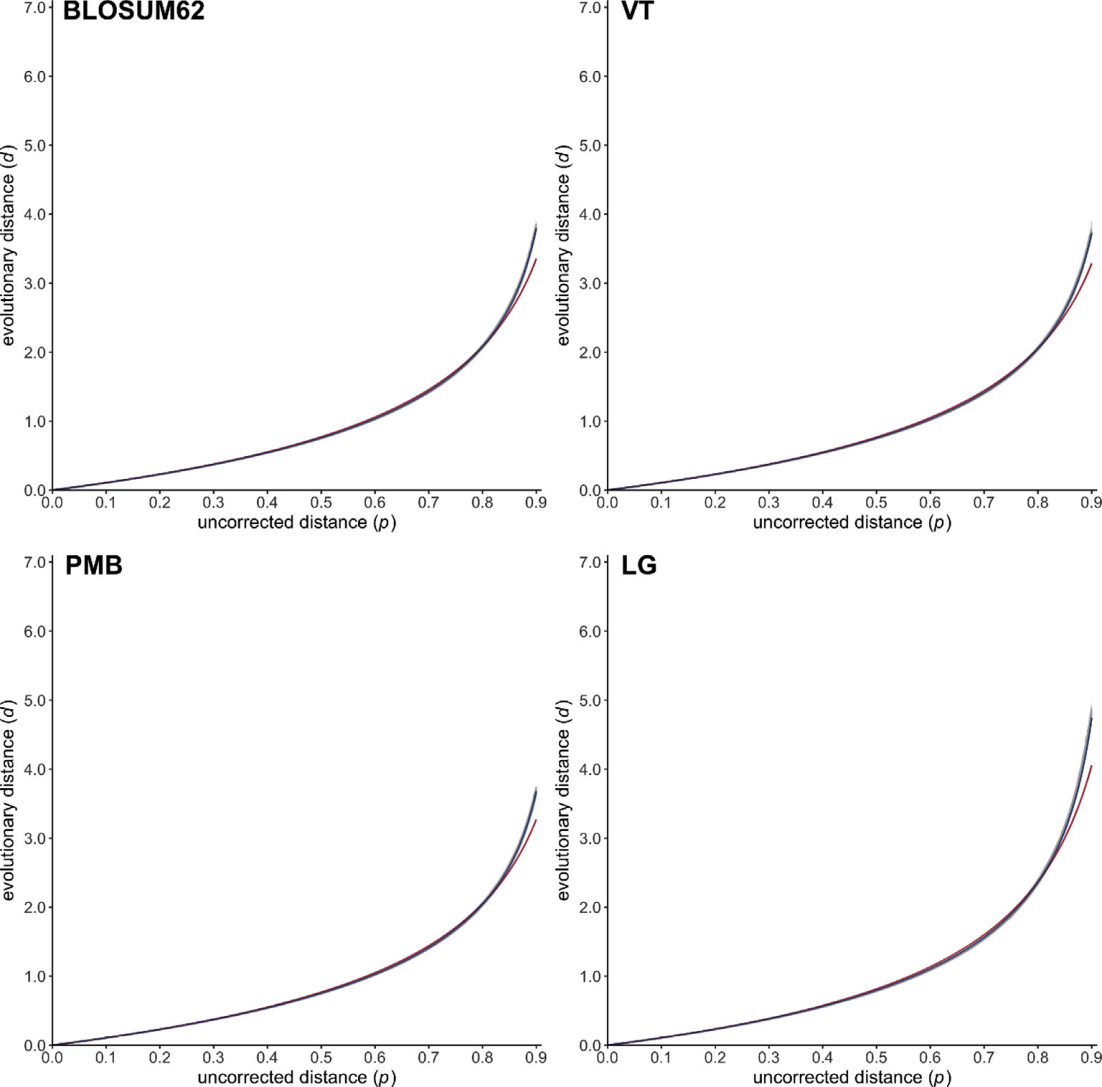
Scatter plots (gray dots) representing the relationship between the uncorrected distance *p* (x-axis) and the evolutionary distance *d* (y-axis) for the four general amino acid substitution models BLOSUM62 [17], VT [22], PMB [25] and LG [29]. Estimated PC and EI gamma distance functions are drawn in red and blue, respectively.

**Fig. 3:**
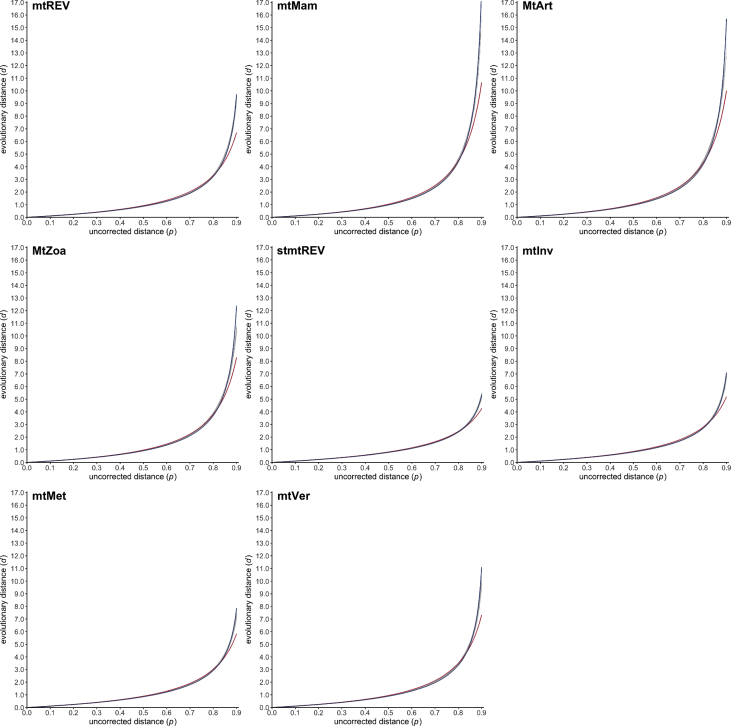
Scatter plots (gray dots) representing the relationship between the uncorrected distance *p* (x-axis) and the evolutionary distance *d* (y-axis) for the eight mitochondrial amino acid substitution models mtREV [19], mtMam [20], MtArt [28], MtZoa [30], stmtREV [34], mtInv, mtMet and mtVer [36]. Estimated PC and EI gamma distance functions are drawn in red and blue, respectively.

**Fig. 4:**
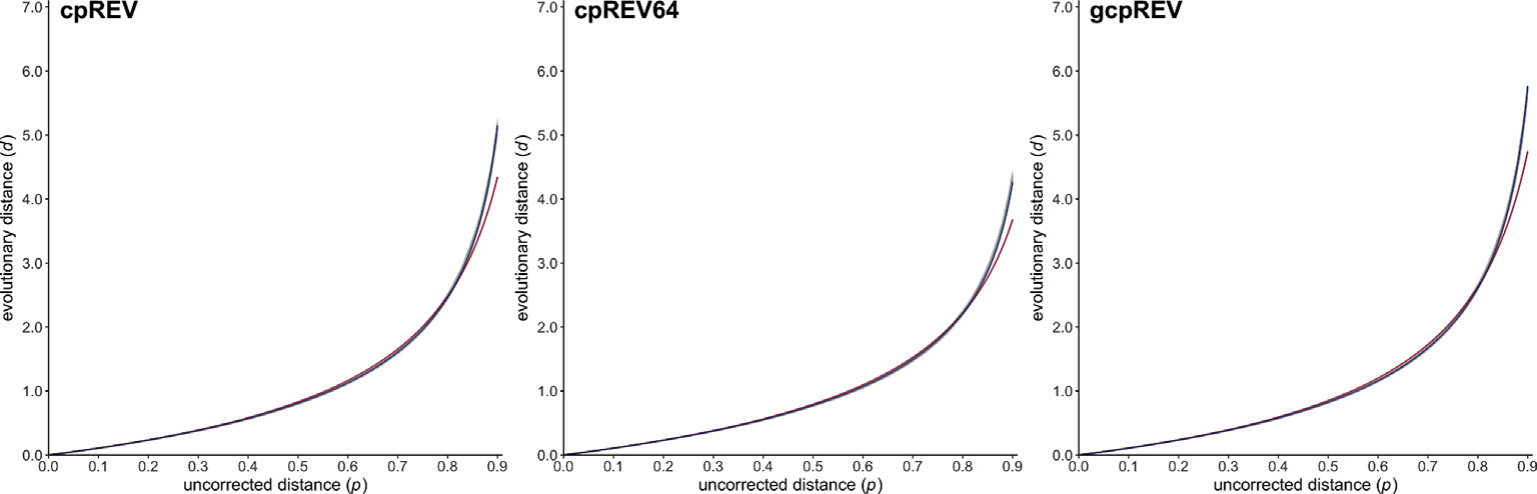
Scatter plots (gray dots) representing the relationship between the uncorrected distance *p* (x-axis) and the evolutionary distance *d* (y-axis) for the three amino acid substitution models cpREV [21], cpREV64 [31] and gcpREV [33] dedicated to plastid-encoded protein sequences. Estimated PC and EI gamma distance functions are drawn in red and blue, respectively.

**Fig. 5:**
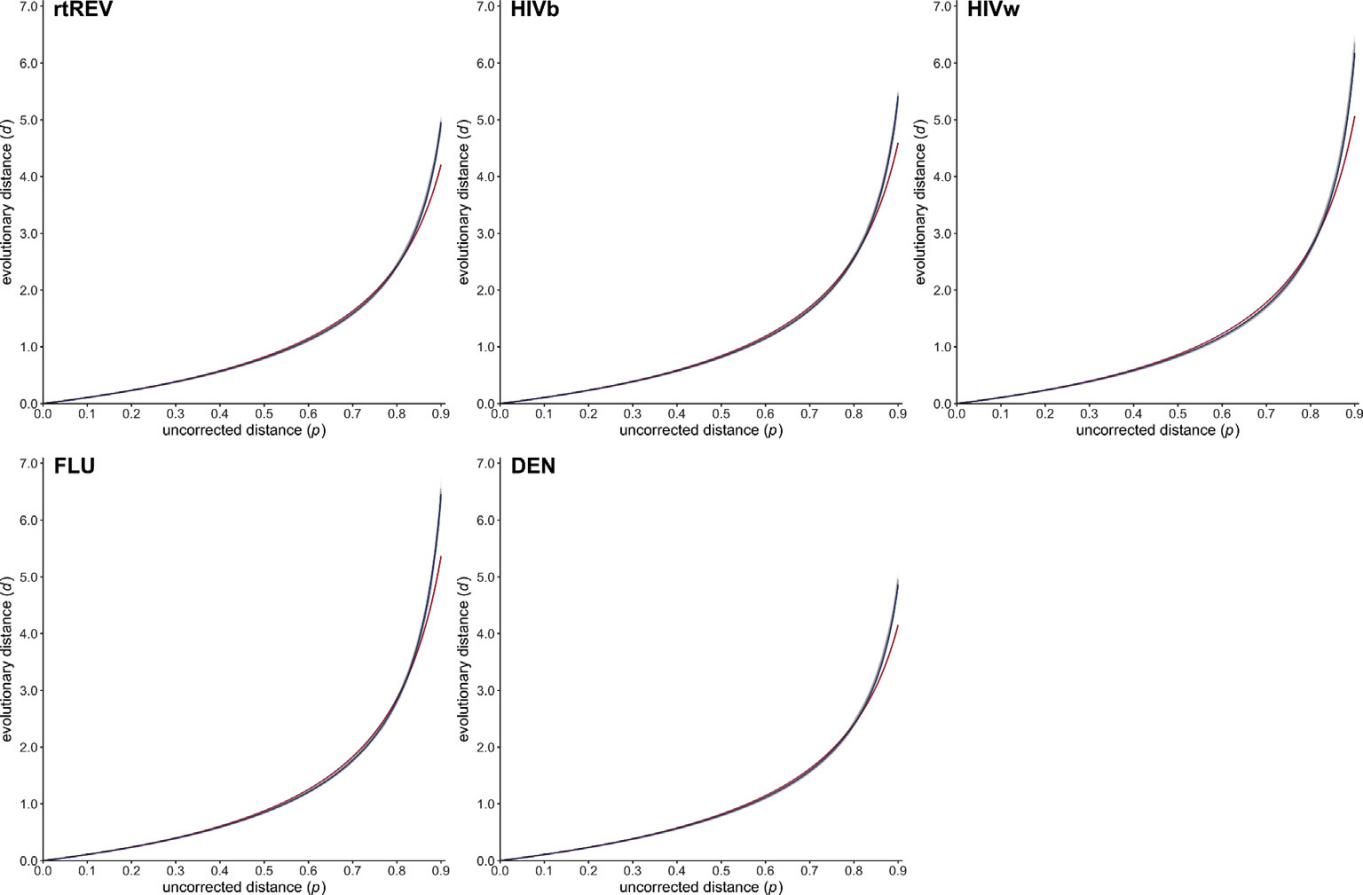
Scatter plots (gray dots) representing the relationship between the uncorrected distance *p* (x-axis) and the evolutionary distance *d* (y-axis) for five amino acid substitution models dedicated to retrovirus (rtREV [24]), HIV (HIVb, HIWw [27]), influenza (FLU [32]), and dengue (DEN [37]) protein sequences. Estimated PC and EI gamma distance functions are drawn in red and blue, respectively.

**Fig. 6:**
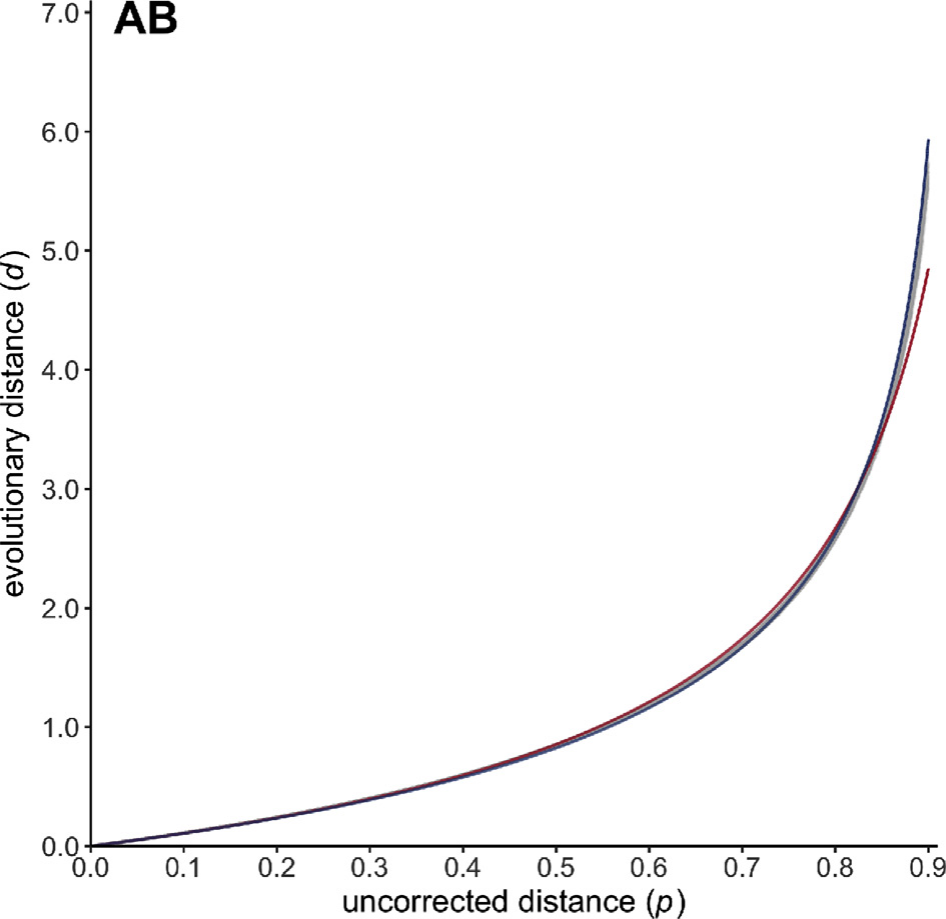
Scatter plot (gray dots) representing the relationship between the uncorrected distance *p* (x-axis) and the evolutionary distance *d* (y-axis) for the antibody-specific model of amino acid substitution AB [35]. Estimated PC and EI gamma distance functions are drawn in red and blue, respectively.

Given a pair of homologous amino acid sequences, there exists a strong positive monotonic relationship between the number *d* of substitution events per site that have occured during their evolution and the proportion *p* of observed differences (often called uncorrected distance or *p*-distance) between the two aligned sequences [1], [2], [3], [4], [5], [6], [7], [8], [9]. For estimating the (unknown) evolutionary distance *d* from the observed value *p*, analytical formulae of the following form (often called gamma distance) have been proposed:

(1) *d = a b* [ (1 − *p* / *b*)^−1/*a*^ − 1 ]

where *a* and *b* are two positive numerical parameters depending on the heterogeneity of the replacement rate among amino acid pairs and sites, and on the equilibrium frequencies of amino acid residues, respectively [2], [10], [11], [12], [13], [14], [15]. In line with previous attempts [2], [4], [5], [14], [16], data presented here are estimations of *a* and *b* as obtained through computer simulations for 27 empirical models of amino acid substitution [1], [17], [18], [19], [20], [21], [22], [23], [24], [25], [26], [27], [28], [29], [30], [31], [32], [33], [34], [35], [36], [37] (see names and associated references in Table 1, Table 2), as well as the associated text files containing simulation datasets (https://zenodo.org/record/2653704) and figures showing the relationship between *p* and *d* (Fig. 1, Fig. 2, Fig. 3, Fig. 4, Fig. 5, Fig. 6, and image files available at https://zenodo.org/record/2653704).

**Table 1:** Poisson correction (PC) gamma distance: estimated values and associated statistics of the numerical constants *a* for 27 empirical models of amino acid substitution.

Evolutionary model	*b*	*a*			
		Estimate	95% confidence interval		Mean squared error
Dayhoff [1]	1.00000	1.99924	1.99850	1.99997	0.00121
BLOSUM62 [17]	1.00000	3.24334	3.24188	3.24481	0.00064
JTT [18]	1.00000	2.57163	2.57057	2.57270	0.00089
mtREV [19]	1.00000	1.23867	1.23812	1.23922	0.00496
mtMam [20]	1.00000	0.90348	0.90324	0.90372	0.00365
cpREV [21]	1.00000	1.98628	1.98556	1.98699	0.00119
VT [22]	1.00000	3.41801	3.41628	3.41975	0.00072
WAG [23]	1.00000	2.69788	2.69665	2.69910	0.00096
WAG* [23]	1.00000	2.80430	2.80305	2.80555	0.00084
rtREV [24]	1.00000	2.08011	2.07936	2.08087	0.00107
PMB [25]	1.00000	3.45924	3.45765	3.46084	0.00059
DCMut-Dayhoff [26]	1.00000	2.01070	2.00996	2.01144	0.00120
DCMut-JTT [26]	1.00000	2.55191	2.55086	2.55295	0.00088
HIVb [27]	1.00000	1.83588	1.83529	1.83646	0.00110
HIVw [27]	1.00000	1.62839	1.62776	1.62902	0.00210
MtArt [28]	1.00000	0.93628	0.93602	0.93653	0.00345
LG [29]	1.00000	2.21046	2.20952	2.21140	0.00129
MtZoa [30]	1.00000	1.05466	1.05439	1.05492	0.00235
cpREV64 [31]	1.00000	2.63503	2.63381	2.63625	0.00103
FLU [32]	1.00000	1.52820	1.52775	1.52865	0.00144
gcpREV [33]	1.00000	1.76147	1.76090	1.76205	0.00128
stmtREV [34]	1.00000	2.03813	2.03719	2.03908	0.00184
AB [35]	1.00000	1.71521	1.71480	1.71562	0.00075
mtInv [36]	1.00000	1.57997	1.57919	1.58076	0.00373
mtMet [36]	1.00000	1.40469	1.40420	1.40518	0.00240
mtVer [36]	1.00000	1.15596	1.15558	1.15634	0.00330
DEN [37]	1.00000	2.12834	2.12753	2.12915	0.00111

**Table 2:** Equal-input (EI) gamma distance: estimated values and associated statistics of the numerical constants *a* and *b* for 27 empirical models of amino acid substitution.

Evolutionary model	*b*	*a*			
		Estimate	95% confidence interval		Mean squared error
Dayhoff [1]	0.93993	3.14582	3.14550	3.14613	0.00005
BLOSUM62 [17]	0.94151	6.32690	6.32599	6.32782	0.00002
JTT [18]	0.94191	4.39688	4.39633	4.39744	0.00004
mtREV [19]	0.92467	1.95601	1.95578	1.95623	0.00024
mtMam [20]	0.92473	1.30527	1.30514	1.30539	0.00040
cpREV [21]	0.93916	3.14971	3.14940	3.15002	0.00005
VT [22]	0.94092	6.96847	6.96714	6.96980	0.00003
WAG [23]	0.94055	4.81653	4.81579	4.81726	0.00005
WAG* [23]	0.94055	5.01598	5.01518	5.01679	0.00005
rtREV [24]	0.94024	3.30578	3.30545	3.30612	0.00005
PMB [25]	0.94195	7.10575	7.10459	7.10691	0.00002
DCMut-Dayhoff [26]	0.93993	3.16983	3.16951	3.17015	0.00005
DCMut-JTT [26]	0.94193	4.36663	4.36607	4.36719	0.00004
HIVb [27]	0.94179	2.77572	2.77550	2.77594	0.00004
HIVw [27]	0.93819	2.45611	2.45584	2.45639	0.00012
MtArt [28]	0.92743	1.35206	1.35186	1.35226	0.00095
LG [29]	0.94051	3.56820	3.56767	3.56873	0.00009
MtZoa [30]	0.92686	1.57251	1.57227	1.57275	0.00068
cpREV64 [31]	0.93948	4.64357	4.64279	4.64436	0.00006
FLU [32]	0.94110	2.22717	2.22704	2.22731	0.00005
gcpREV [33]	0.93745	2.72778	2.72755	2.72800	0.00005
stmtREV [34]	0.92778	3.77358	3.77322	3.77395	0.00004
AB [35]	0.93407	2.78549	2.78473	2.78625	0.00058
mtInv [36]	0.92211	2.85866	2.85835	2.85897	0.00011
mtMet [36]	0.92546	2.34419	2.34387	2.34451	0.00024
mtVer [36]	0.92052	1.91274	1.91241	1.91307	0.00067
DEN [37]	0.94143	3.34672	3.34632	3.34712	0.00006

## 2 Experimental design, materials, and methods

To simulate the evolution of amino acid sequences along reliable real-case phylogenetic trees, the 1,903,844 available ones on the ftp repository of PhylomeDB v4 (ftp://phylomedb.org/phylomedb) were considered, as they have been inferred by a workflow including homologous sequence clustering and alignment from a broad range of genes and phyla (eukaryota, bacteria and archaea; see details at http://phylomedb.org) followed by maximum likelihood phylogenetic inference [38]. A reduced subset of these trees was built to obtain a wide array of induced patristic distances that are quite evenly distributed over [0, 20] (see y-axis ranges in Fig. 1, Fig. 2, Fig. 3, Fig. 4, Fig. 5, Fig. 6): for *m* growing from 0.0001 to 20 (step = 0.001), one tree (at least 25 taxa) was picked out such that its diameter (i.e. maximum patristic distance) was as close as possible to *m*. Following this procedure, 20,000 real-case phylogenetic trees representative of a comprehensive range of evolutionary events and distances were selected. For each considered evolutionary model (see Table 1, Table 2), the evolution of a sequence of 50,000 amino acid residues was simulated using INDELible v1.03 [39] along each of the 20,000 selected phylogenetic trees, and the matrix of observed *p*-distances was computed from each of the simulated multiple sequence alignments using FastME v2.1.5 [40]. Next, for each evolutionary model, a subset of simulated data (i.e. phylogenetic tree, simulated multiple sequence alignment, and corresponding *p*-distance matrix) was selected to obtain at least 500,000 values *p* that approximately follow a uniform distribution over [0, 0.9]. For each of those selected multiple sequence alignments, the branch lengths of the associated phylogenetic tree were refitted using RAxML-NG v0.8.1 BETA [41] with the corresponding evolutionary model, and the matrix of patristic distances *d* was computed using gotree v0.2.10 (https://github.com/evolbioinfo/gotree). Of note, for each of the 27 considered evolutionary models, INDELible and RAxML-NG were both used with the corresponding empirical replacement matrix file gathered from http://giphy.pasteur.fr/empirical-models-of-amino-acid-substitution. Finally, as each pair of distance matrices (i.e. uncorrected and evolutionary distances *p* and *d*) represents numbers of observed differences and occurred substitutions per site, respectively, each entry was multiplied by the total number of sites (i.e. 50,000) and rounded to the closest integer. This scaling and rounding step allows observing the same integer values than the ones obtained with alternative programs for branch length refitting (e.g. PhyML [42], IQ-TREE [43]) while each program leads to slightly different evolutionary distances *d* because of rounding errors or implementation choices (not shown).

Two versions of the nonlinear functional relationship between the evolutionary distance *d* and the uncorrected distance *p* were fitted separately to each simulated data. The first, called the Poisson correction (PC) gamma distance, is determined by fixing *b* = 1 in formula (1) [2], [5], [44]. The second, called the equal-input (EI) gamma distance, is determined with *b* = 1 − ∑_*r*_ *π*_*r*_^2^ in formula (1), where *π*_*r*_ is the equilibrium frequency of the amino acid residue *r* [12], [13], [15]. For each of the 27 considered evolutionary models, empirical values of *π*_*r*_ from the corresponding amino acid replacement matrix were used for computing *b* (Table 2). For each evolutionary model and each of the two PC and EI gamma distances, the numerical constant *a* was estimated by weighted nonlinear regression from the pairs of integer versions of uncorrected and evolutionary distances *p* and *d* gathered from the corresponding simulation file (see above) and divided by the number of simulated sites (i.e. 50,000). Each least-square estimation of the parameter *a* was performed using R v3.5.3 [45] with the function nls. Default Gauss-Newton algorithm was used with relative weighting (i.e. each *d* was weighted with *d*^−2^) and starting value *a* = 2.

All simulation datasets are available at https://zenodo.org/record/2653704 (doi:10.5281/zenodo.2653704). The 20,000 phylogenetic trees selected for simulating sequence evolution are available as a text file together with descriptive statistics summarizing the corresponding patristic distances. For each of the 27 evolutionary models (see Table 1, Table 2), blocks of simulation data (i.e. PhylomeDB identifiers, random seeds, trees with refitted branch lengths, integer values of *p* and *d*) are available as text files. Estimated values of *a* are given for the PC and EI gamma distances in Table 1, Table 2, respectively, together with the associated 95% confidence intervals and mean squared errors. Fig. 1, Fig. 2, Fig. 3, Fig. 4, Fig. 5, Fig. 6 represent the 27 scatter plots of simulated *d* against *p*, as well as the regression curves for the two PC and EI gamma distance functions. Each scatter plot is also available with and without the regression curves at https://zenodo.org/record/2653704.

## Funding

This research did not receive any specific grant from funding agencies in the public, commercial, or not-for-profit sectors.
